# Impact of preoperative Vitamin D_3_ administration on postoperative hypocalcaemia in patients undergoing total thyroidectomy (HypoCalViD): study protocol for a randomized controlled trial

**DOI:** 10.1186/s13063-016-1216-5

**Published:** 2016-02-20

**Authors:** Stefanie Wolak, Mandy Scheunchen, Katharina Holzer, Mirjam Busch, Esra Trumpf, Andreas Zielke

**Affiliations:** Sana-Klinikum Offenbach, Chirurgische Klinik, Starkenburgring 66, 63069 Offenbach am Main, Germany; Universitätsklinikum der Goethe-Universität Frankfurt, Fachbereich Zahnmedizin, 60590 Frankfurt, Germany; Diakonie-Klinikum Stuttgart, Klinik für Endokrine Chirurgie, Endokrines Zentrum Stuttgart, Rosenbergstrasse 38, 70176 Stuttgart, Germany; Universitätsklinikum der Goethe-Universität Frankfurt, Klinik für Allgemein- und Viszeralchirurgie, 60590 Frankfurt, Germany

**Keywords:** calcitriol, hypocalcaemia, hypoparathyroidism, total thyroidectomy, vitamin D deficiency

## Abstract

**Background:**

Total thyroidectomy is increasingly used as a surgical approach for many thyroid conditions. Subsequently, postoperative hypocalcaemia is observed with increasing frequency, often resulting in prolonged hospital stay, increased use of resources, reduced quality of life and delayed return to work. The administration of vitamin D is essential in the therapy of postoperative hypocalcaemia; calcitriol is most commonly used. What has not been examined so far is whether and how routine preoperative vitamin D prophylaxis using calcitriol can help to prevent postoperative hypocalcaemia. This study evaluates routine preoperative calcitriol prophylaxis for all patients who are to undergo a total thyroidectomy, compared with the current standard of post-treatment, i.e., selective vitamin D treatment for patients with postoperative hypocalcaemia.

**Methods/design:**

This clinical observational (minimal interventional clinical trial) trial is a multicentre, prospective, randomized superiority trial with an adaptive design. Datasets will be pseudonymized for analysis. Patients will be randomly allocated (1:1) to the intervention and the control groups. The only intervention is 0.5 μg calcitriol orally twice a day for 3 days prior to surgery. For the primary endpoint measure (number of patients with hypocalcaemia), hypocalcaemia is defined as serum calcium of less than 2.1 mmol/l on any day during the postoperative course; this measure will be analyzed using a Chi-square test comparing the two groups. Secondary endpoint measures, such as number of days to discharge, quality of life, and economic parameters will also be analyzed.

**Discussion:**

By virtue of the direct comparison of clinically and economically relevant endpoints, the efficacy as well as efficiency of preoperative calcitriol prophylaxis of hypocalcaemia will be clarified. These results should be available 24 months after the first patient has been enrolled. The results will be used to inform a revised practice parameter guideline of whether or not to recommend preoperative calcitriol for all patients in whom total thyroidectomy is planned.

**Trial registration:**

Deutsches Register Klinischer Studien, DRKS00005615 (Feb.12.2016).

## Background

Surgery of the thyroid gland is one of the five most common surgical procedures in Germany, accounting for some 90000 operations per year [1]. Because subtotal thyroidectomy is no longer regarded the treatment of choice for multinodular goitre and Graves’ disease [2], the number of patients treated by total thyroidectomy has progressively increased, amounting to 47.9 % in 2011 [3]. This surgical approach aims to prevent recurrent disease and is supported by current guidelines, such as the national S2-guideline issued by the Work Group of the Scientific Medical Professional Societies [4]. However, postoperative symptomatic hypocalcaemia due to transient hypoparathyroidism is a frequent condition after total thyroidectomy, and is reported to occur in up to 54 % of patients [5]. Owing to the extent of resection in the case of total thyroidectomy, the viability of all four parathyroid glands may be endangered because of surgical trauma, and their function may be temporarily or permanently impaired. Risk factors for transient or permanent hypoparathyroidism after total thyroidectomy are higher age, female sex, Graves’ disease, need for parathyroid autotransplantation, inadvertent excision of parathyroid glands and low postoperative parathyroid hormone and serum calcium levels, as well as low preoperative serum 25-hydroxy vitamin D level [6].

Postoperative hypocalcaemia affects patients’ physical health and quality of life and causes a significant economic burden for the healthcare system. Acute hypocalcaemia may cause seizures or cardiac or digestive dysfunction, as well as phobic psychological disorders. Hypocalcaemic patients require continuous medical attention, frequent and more extensive biochemical tests and more medical treatment, including more medication. Moreover, a recent observational study reported the average length of hospital treatment of patients with postoperative hypocalcaemia to be increased by 2.2 days, compared with that of normocalcaemic patients [5]. Hence, the search for methods to reduce the incidence and severity of postoperative hypocalcaemia is of clinical, medical and economic relevance.

Despite ongoing research on the prevention of postoperative hypocalcaemia, its treatment is well established and uses oral calcium and vitamin D supplementation. Based on empirical evidence, Tartaglia *et al.* [7] were the first to show that routine postoperative administration of calcitriol and calcium salts significantly decreased the risk of severe postoperative hypocalcaemia. Indeed, low levels of vitamin D, as determined by preoperative testing, were shown to be an independent risk factor for postoperative hypocalcaemia [8], suggesting that correction of low vitamin D levels might be a useful measure to reduce postoperative hypocalcaemia rates.

Vitamin D metabolites and analogues are essential in the management of postsurgical hypoparathyroidism. The active form of vitamin D – calcitriol (1,25-dihydroxyvitamin D_3_) – is the preferred clinical option because of its potency and rapid onset and offset of action. Calcitriol regulates the expression of TRPV6, which is a calcium entry channel responsible for calcium absorption in the intestine [9], so that there is a time of onset of action of only some 1 or 2 days compared with 10–14 days for Vitamin D_3_ (cholecalciferol) [10].

We therefore hypothesized that preoperative administration of calcitriol would reduce the rate of hypocalcaemia after total thyroidectomy. Moreover, if postsurgical hypocalcaemia should occur, preoperative vitamin calcitriol prophylaxis should be helpful in reducing the time to manage this condition and thus the duration of hospital stay. So far, and to the best of our knowledge, studies to that effect are still missing.

### Aim of the study

The overall aim of this study is to evaluate the effect of an early onset of calcitriol supplementation on postoperative hypocalcaemia. This study is specifically designed to evaluate the effects of a preoperative short term administration of calcitriol prior to total thyroidectomy (intervention) in comparison with current standard clinical protocols of postoperative administration of calcitriol in only those patients who have postoperative hypocalcaemia (control group). Calcitriol will be administered according to well-established clinical protocols and no additional diagnostic interventions are intended, in order to reduce the likelihood of adverse events. This study is therefore planned as a post-marketing noninterventional, i.e., observational study. Although we consider HypoCalViD to be an observational trial rather than a prospective randomized controlled trial, because patients are randomly allocated to an interventional and a noninterventional arm prior to surgery, this trial may be attributed a ‘minimal-intervention clinical trial’ as recently defined by EU-directive number 536/2014 of 16 April 2014.

## Methods/design

### Trial population and eligibility criteria

**Fig. 1:**
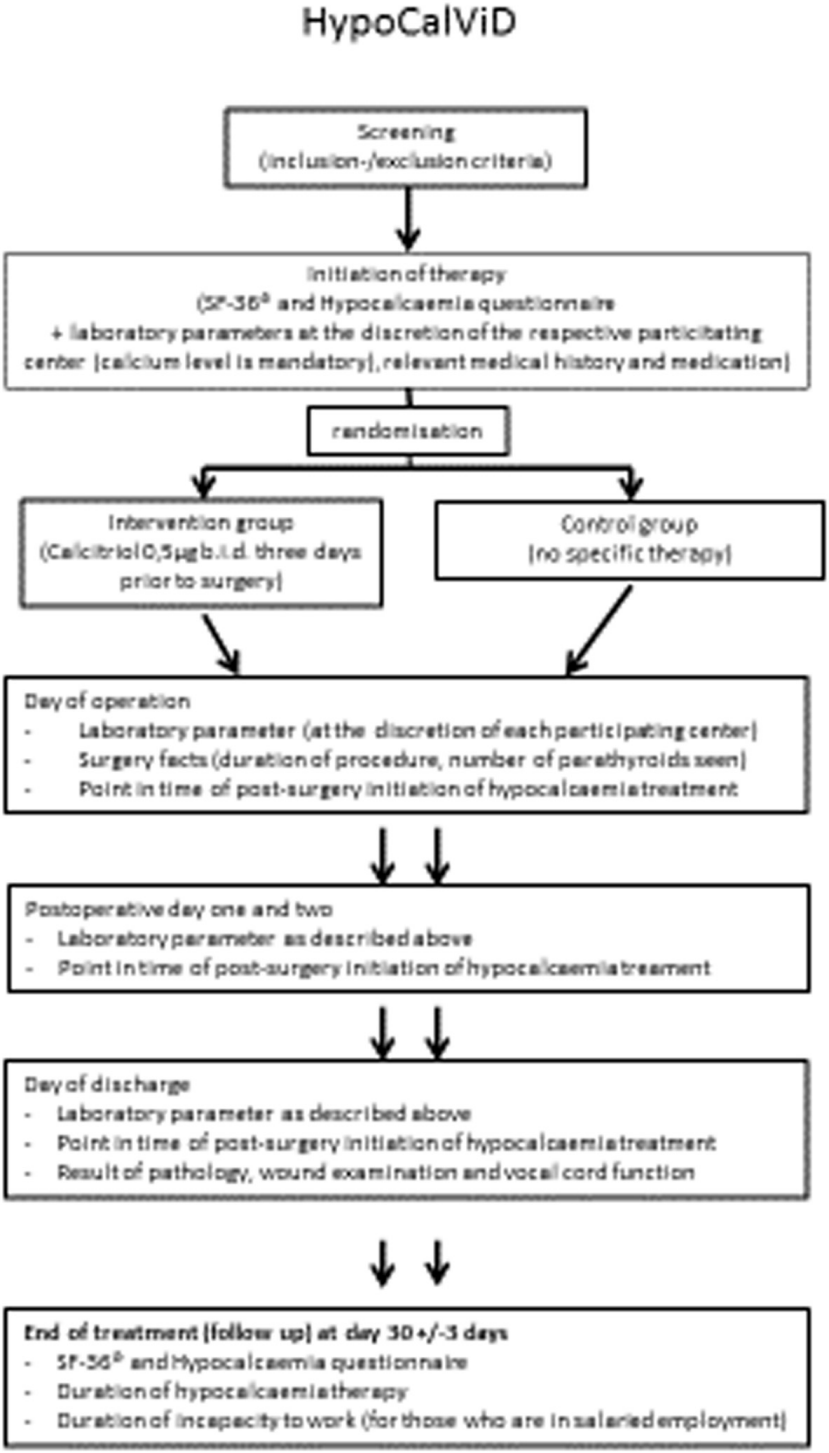
Setup of the study

HypoCalViD is a prospective, randomized, multicentre study (Fig. 1) involving patients with benign thyroid disease (Graves’ disease, multinodular goitre, hyperthyroidism) undergoing primary elective total thyroidectomy. Such patients will be eligible to participate after they have given their written informed consent. Patients with conditions that could potentially affect serum calcium levels, such as musculoskeletal diseases, hyperparathyroidism and medications known to affect calcium metabolism are excluded.

This multicentre study will be conducted in hospital departments with the required staffing levels and structural as well as scientific volume qualifications. Only hospital departments with a special interest in endocrine surgery and a working volume accounting for more than 100 total thyroidectomies per year, will be eligible to participate. The head surgeons of these departments must be members of the German Association of Endocrine Surgeons of the Deutsche Gesellschaft für Allgemein und Viszeralchirurgie.

### Sample size

The sample size has been calculated based on assumptions generated from a critical appraisal of current evidence. According to prior publications, 25 % of the patients in the control group can be expected to develop postsurgical hypocalcaemia as compared with 15 % of the patients in the intervention group. Assuming an α-level of 5 % and a power of 80 %, it is calculated that 500 patients must be recruited to obtain significant results. Assuming a cumulative drop-out rate of 10 %, 700 patients will have to be screened in order to obtain a total number of 600 patients for random allocation. Subsequently, it is expected that data obtained from 540 patients will be analyzed at primary endpoint. By using an adaptive study design, the number of patients will be re-evaluated after the first 270 patients have been treated, with complete follow-up.

#### Feasibility

A small number of high-volume providers will be eligible to participate in this study. As this study will take place only at high-volume centres chaired by highly specialized endocrine surgeons, we assume that the operation will be done or supervised by a specialist. Stratification, therefore, is done by centres rather than by surgeon. In 2013, these high-volume providers took care of some 2500 surgical cases. All of the potential study centres are active members of the German Association of Endocrine Surgeons and have been involved in clinical multicentre trials in the past. Given these facts, it was reasonable to assume the average recruitment rate with a minimum of 60 patients per year and per centre, resulting in an estimated recruitment phase of 24 months.

### Type of trial

This study is designed as a multicentre, parallel-designed, randomized, clinical, observational trial (minimal-intervention clinical trial).

### Randomization

Randomization to the treatment arm and the control group will be performed in a 1:1 ratio, stratified by each participating centre. Random allocation of patients will be dynamical. For the randomization procedure, the computer program ‘Random Allocation Software’ (Version 1.0.0) will be used; permuted block sizes will be 30 and 60.

### Intervention

The specific trial intervention consists of 0.5 μg calcitriol orally, twice a day for 3 days prior to surgery. The control therapy consists of any standard conventional hypocalcaemia therapy according to current guidelines or local clinical standards of the participating clinical centre and adherent to the implemented standards of the respective institution. Standard therapy is defined as the currently accepted and used treatment for this condition, based on the results of past research.

### Endpoint measures

#### Primary endpoint measure

The incidence of postsurgical hypoparathyroidism is defined as the primary endpoint measure. Postsurgical (transient) hypoparathyroidism is defined as the number of participants with a postoperative serum calcium concentration <2.1 mmol/l on any day of the postoperative hospitalization period. Since there is no evidence- or consensus-based definition of postoperative hypoparathyroidism based on calcium and parathyroid hormone cutoff levels, we defined postoperative hypoparathyroidism as calcium and parathyroid hormone levels below the normal range (Ca < 2.1 mmol/l), in line with expert opinion [11]. Application type, form and dosage of calcium and vitamin D administration therapy to achieve normocalcaemia will be recorded. Study parameters will be recorded before surgery, on postoperative days 1 and 2, and at discharge, as well as 30 ± 3 days following surgery.

#### Secondary endpoint measures

Secondary endpoint measures comprise the overall duration of hospital stay, the number of days until restoration of normal serum calcium level, and hospital resource utilization, as well as quality of life and reported patient outcomes.

Duration of hospital stay will be documented according to hospital charts. Prior to and one month after surgery, patients will be asked to complete the well-established SF36 Quality of Life questionnaire [12] and a questionnaire constructed for the assessment of postoperative hypocalcaemia [13]. The number of days needed to adjust hypocalcaemia to a normal level (calcium level = 2.2 to 2.7 mmol/l) will also be assessed.

#### Safety endpoint measures

The incidences of serious adverse events, drug-related adverse events and mortality from any cause (within 30 days of the time of initiation of therapy) are considered safety endpoint measures of this trial.

#### Economics-based outcome measures

Direct resource use defined by the number of days of in-patient hospital treatment will be documented. Direct medical resource use (direct medical costs) includes the resource use that is an immediate consequence of the disease pattern, such as general and specific clinical diagnostics, medication (in particular vitamin D analogues and calcium supplements), secondary interventions and readmissions. Direct nonmedical resource use is the resource use caused by consequences arising from the postoperative condition, namely the incapacity to work for those who are otherwise in salaried employment.

### Data analysis

The primary hypothesis is that the rate of postoperative hypocalcaemia is lower in the preoperative vitamin D intervention group than in the standard postoperative treatment control group. The secondary hypothesis postulates statistically significant clinical and health economic benefits through the intervention, as compared with the current standard treatment.

The primary effectiveness parameter will be analyzed using a Chi-square test comparing the two groups. Supportive statistical analysis will include logistic regression with factors in the model to include treatment, study centre, age, sex and vitamin D status (appropriate baseline parameters and medical history information may also be considered). The secondary efficacy parameter will be compared between the two treatment groups using a log rank test. In addition, as mentioned, a supportive analysis making use of a Cox proportional hazards regression will also be used, to assess whether there is a difference between treatment groups while accounting for the effect of study centre and other appropriate baseline and medical history parameters. A planned interim analysis will be performed when 270 study participants have completed the study.

The interim analysis will re-evaluate the assumptions made for the study sample size calculation; at this point, the sample size will be re-estimated unless the trial has to be stopped as a result of the interim analysis.

### Trial organization, registration and ethical aspects

To ensure data integrity and unbiased clinical decisions, the steering committee will make use of a data management plan. This includes regular visits of all trial sites for source data verification, to ensure continuous monitoring of the study data for completeness, validity and plausibility. During those visits, random samples of participants’ files will be monitored to verify accurate data registration and management.

The trial will be performed according to the Declaration of Helsinki in its current version and Good Clinical Practice guidelines. Before the start of the trial, the independent ethic committee of the Landesärztekammer Hessen gave a positive vote on the 16 July 2013: the committee stated that there were no more ethical and juridical doubts against the implementation of the trial.

The trial is registered with the Deutsches Register Klinischer Studien [14], number DRKS00005615.

## Discussion

Total thyroidectomy is currently the standard surgical procedure for various conditions of the thyroid gland; the aim is to reduce the incidence of recurrent disease and thus avoid re-operations. As a consequence of the more extensive surgical procedure, postoperative hypoparathyroidism is affecting an increasing number of patients. In addition, it has become a burden for the health system, because hypocalcaemic patients require longer in-patient treatment and more medical resources. HypoCalViD has the potential to prove the clinical usefulness of a rather simple intervention with a well-established protocol, commonly used for the correction of postsurgical hypocalcaemia: calcitriol.

A potential weakness of the protocol is that there is neither the patient nor the investigator is blinded. However, as the primary endpoint (serum calcium concentration) is measured using standardized methods and independently of the respective intervention, a placebo effect is unlikely to occur. Secondary endpoint measures, such as the score on the SF36 questionnaire addressing the participants’ quality of life, might also be influenced by the patients’ awareness of having been part of the intervention or control group.

Because this trial aims to address the most common thyroid procedure, i.e., thyroidectomy for benign thyroid conditions, comprising more than 80 % of thyroid procedures, nonbenign reasons for total thyroidectomy were excluded. Patients with malignant conditions often require more extensive resections and might experience hypocalcaemia more often. There might be an even greater effect of calcitriol in these cases. However, these cases only make up for some 5–10 % of all thyroid procedures and the extent of surgery is tailored to the individual patient, making it difficult to study such a heterogenous group.

To obtain a comparable group of postsurgical patients with the same extent of surgical trauma and because a large number of patients is needed to be able to demonstrate a significant effect of the intervention, we decided to exclude malignant deceases for the specific purpose of this study.

Calcitriol will be given to the patients regardless of their vitamin D status, although prior evidence suggests that including patients only with a documented low vitamin D level would potentially increase the likelihood of a positive effect of calcitriol administration (the intervention) on both the primary and secondary outcome parameters. The main reason for not taking preoperative vitamin D levels into account is that this study aims at not interfering with the current standard of care or established guideline-oriented clinical management of patients with postoperative hypocalcaemia after thyroid surgery.

Although the assessment of vitamin D levels is recommended by guidelines addressing common problems of older people, such as osteoporosis, routine assessment of vitamin D is not yet established in clinical practice in Germany. In addition, determination of a patient’s vitamin D level incurs a minimum charge of €27.98 – with a potential for additional costs of €2.60 for taking the blood sample – while the calcitriol prophylaxis employed in this study proposal incurs an additional cost of less than €5 per patient. Because such a routine prophylaxis is considerably less costly and – given a positive outcome of this study – much easier to implement, the trial is likely to offer a realistic chance of optimizing treatment of patients undergoing thyroid surgery.

The primary endpoint measure is defined by total serum calcium level. Although ionized calcium level might be more specific in detecting hypocalcaemia, especially in the presence of malnutrition, ionized calcium is not a commonly used laboratory parameter during postsurgical follow-up of thyroid patients. We decided to ask for total calcium level only, as most hospitals and surgical departments make use of this parameter. Moreover, the observational nature of this trial intends not to interfere with the respective standard of treatment. Therefore, no additional parameters other than the individual trial sites’ ‘usual’ parameters were included.

Moreover, the majority of patients with elective benign thyroid surgery should not suffer from severe protein deficiency.

By virtue of the direct comparison of clinically and economically relevant endpoints, this trial should assist in clarifying the efficacy as well as efficiency of preoperative calcitriol prophylaxis for patients undergoing total thyroidectomy. First results should be available 24 months after the first patient has been enrolled. The results will be used as a rationale to propose a revised practice parameter guideline to the CAEK-DGAV (Chirurgische Arbeitsgemeinschaft Endokrinologie, Deutsche Gesellschaft für Allgemein- und Viszeralchirurgie) of the German Association of Surgeons, whether or not to recommend general preoperative calcitriol supplementation for patients in whom a total thyroidectomy is planned.

## Trial status

Up to now, six centres are initiated and are actively recruiting patients. The first patient was enrolled on 4 July 2014. The expected recruitment period will last until July 2016.

## Abbreviation

CAEK-DGAV: Chirurgische Arbeitsgemeinschaft Endokrinologie, Deutsche Gesellschaft für Allgemein- und Viszeralchirurgie
